# Dual-Emissive Rectangular Supramolecular Pt(II)-*p*-Biphenyl with 4,4′-Bipyridine Derivative Metallacycles: Stepwise Synthesis and Photophysical Properties

**DOI:** 10.3390/molecules28217261

**Published:** 2023-10-25

**Authors:** Antonia Garypidou, Konstantinos Ypsilantis, John C. Plakatouras, Achilleas Garoufis

**Affiliations:** 1 Department of Chemistry, University of Ioannina, GR-45110 Ioannina, Greece; a.garypidou@uoi.gr (A.G.); k_ipsilantis@yahoo.gr (K.Y.); iplakatu@uoi.gr (J.C.P.); 2 Institute of Materials Science and Computing, University Research Centre of Ioannina (URCI), GR-45110 Ioannina, Greece

**Keywords:** platinum(II), supramolecular coordination complexes, stepwise synthesis, aggregation-induced emission

## Abstract

Mixed-ligand tetranuclear supramolecular coordination complexes (SCCs) of Pt(II)-*p*-biphenyl and bridging ligands derivatives of 4,4′-bypiridine (**8**)–(**10**), were synthesized and characterized. The SCCs were synthesized stepwise, starting from the Pt-*p*-biphenyl -Pt core. The crystal structure of complex {[Pt(2,2′-bpy)]_4_(*μ*-bph)_2_(*μ*-(4,4′-bpy)_2_}{PF_6_}_4_ (2,2′-bpy = 2,2′-bipyridine, bph = *p*-biphenyl and 4,4′-bpy = 4,4′ bipyridine), was determined using single-crystal diffraction methods. The emission profile of the tetranuclear complexes (**8**)–(**10**) was influenced by the length of the bridging ligands and was found to depend on solvent polarity. Dual-emission patterns in methanol–water mixtures were observed only in the cases of complexes (**9**) and (**10**), attributed to aggregation-induced emission phenomena.

## 1. Introduction

The adjustment of the optical characteristics of transition metal complexes by means of a systematic modification of both their structure and ligands constitutes a significant undertaking within the realm of synthetic inorganic chemistry. Without a doubt, it certainly contributes to the improvement of technological applications, including solar cells [1,2], light-emitting diodes [3,4], nonlinear optical materials (NLO) [5,6], agents for bioimaging and biosensing [7,8], etc., alongside facilitating the systematic investigation of their structure–activity relationship. In recent decades, there has been remarkable research interest regarding the investigation of the optical properties displayed by supramolecular coordination complexes (SCCs), such as 2D-metallacycles and 3D-metallacages. This increased interest can be attributed to their ability to modulate their size, shape, and charge through the coordinated ligands [9].

**Figure 1:**
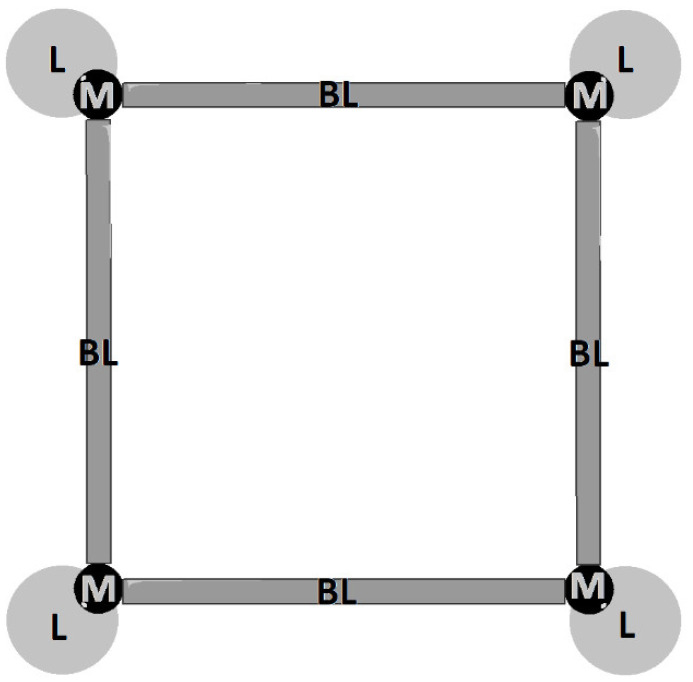
Schematic representation of a 2D metallacycle. M = Pt(II) or Pd(II), BL = Bridging ligand, L = ancillary ligand.

The efficient construction of SCCs can be achieved through either spontaneous coordination-driven self-assembly or through a planned stepwise synthesis. Platinum and palladium molecular squares constitute a significant category of 2D metallacycles, wherein four metal atoms occupy the vertices of a square and are bridged by four bridging ligands. The coordination sphere of each metal is completed by two atoms of ancillary ligand(s) (Figure 1).

The spontaneous formation of tetranuclear square metallocycles through the self-assembly of corresponding square planar metal chelates was initially demonstrated in the early 1990s by Fujita et al. [10]. This pioneering work reported on the synthesis of a palladium tetranuclear complex, namely [Pd(en)(4,4′-bpy)]_4_(NO_3_)_8_, derived from the Pd(en)(NO_3_)_2_ and 4,4′-bpy. The spontaneous self-assembly facilitates the formation of metallacycles wherein all four sides of the square possess identical bridging ligands (BL). This approach is particularly advantageous for palladium complexes, given their greater kinetic lability compared to platinum ones. On the other hand, the synthetic approach of stepwise synthesis of 2D metallacycles was initially introduced by P. Stang. This approach offers the advantage of forming non-symmetric rectangular shapes or mixed metal squares [11]. Additionally, it allows the isolation of intermediate complexes including binuclear complexes, π-shaped complexes, etc. Numerous reports have focused on the synthesis, characterization, and investigation of platinum tetranuclear molecular squares [12,13], all four sides of which are equal in length, since a single type of BL is used. The most-used BLs include 4,4′-bpy [14,15,16] or 4,4′-bpy-like ligands [17,18,19,20,21,22], *p*-biphenyl and its derivatives [23,24,25,26,27]. However, the synthesis of molecular squares using alternative BLs such as pyrazines [28,29], benzonitrile [11], dyalkynyl [30,31], porphyrins [32], ditopic perylene [33], pyrido-isoquinoline derivatives [34], and BODIPY (boron-dipyrromethene)-based ligands [35] etc. has also been reported. In terms of auxiliary ligands, chelated biphosphines and ethylenediamine are commonly used. 

Apart from the rectangular squares, there is another category of supramolecular complexes referred to as rhomboidals. While the angles in the squares are approximately 90°, in rhomboidals, the angles are estimated to be greater due to the *trans*- orientation of bridging ligands [36,37]. Rhomboidal Pt(II) complexes have been reported, starting from 2,9-bis[*trans*-Pt(PEt_3_)_2_NO_3_]phenanthrene and the equivalent addition of a non-linear ligand. These complexes exhibited excellent photophysical properties [37], leading to their potential use in cancer cell imaging and cancer chemotherapy. In vivo experiments have shown low toxicity in healthy cells [38]. Pollock et al. [39] reported on multinuclear bis(phosphine)Pt(II) metal complexes in self-assembled coordination cages due to their favorable photophysical properties, including tunability and long-lived excited states. The focus was on the development of highly emissive rhomboidal structures using aniline-containing donors and Pt-based metal acceptors. The authors successfully synthesized a series of rhomboidal complexes with tunable emission wavelengths across the visible spectrum by varying the functional groups attached to the aniline core. 

**Figure 2:**
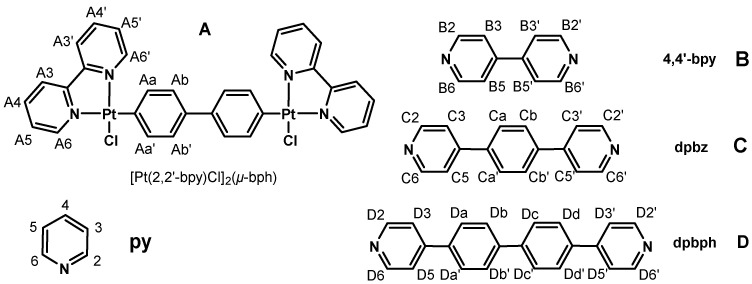
The numbering of structures for the bimetallic complex [Pt(bpy)Cl]_2_(*μ*-bph), (**A**) and the ligands, 4,4′-bipyridine, (**B**), 1,4-di(pyridin-4-yl)benzene, (**C**), and 4,4′-di(pyridin-4-yl)-1,1′-biphenyl, (**D**), used in this study.

While there is extensive literature on the synthesis of various platinum tetranuclear molecular squares with equal in length BLs, the exploration of rectangular structures with different length BLs is relatively rare [21,27]. However, recently Chen et al. [40] reported a series of SCCs with ligands, modified 4,4-bipyridine and *p*-phthalic acid in order to investigate their photophysical properties. The introduction of platinum atoms led to a red-shift in both the absorption and emission spectra when compared to the spectra of the free ligands. Achieving such structures typically involves a stepwise synthesis approach, which we have employed in constructing of the rectangular structures described in this study. More particularly, we synthesized and characterized a series of platinum rectangular metallocycles based on the bimetallic complex [Pt(bpy)Cl]_2_(*μ*-bph), (A), incorporating the 4,4′-bpy-like ligands 4,4′-bipyridine, (B), 1,4-di(pyridin-4-yl)benzene, (C), and 4,4′-di(pyridin-4-yl)-1,1′-biphenyl, (D), each with increased lengths (Figure 2). To promote the aggregation of SCCs, we introduced 2,2′-bipyridine as auxiliary ligand, taking advantage of its ability to facilitate intermolecular stacking between the platinum molecular squares. The optical characteristics of these complexes were investigated, particularly in relation to the solvent polarity and their aggregation degree [41].

## 2. Results and Discussion

### 2.1. Synthesis and Characterization

**Scheme 1:**
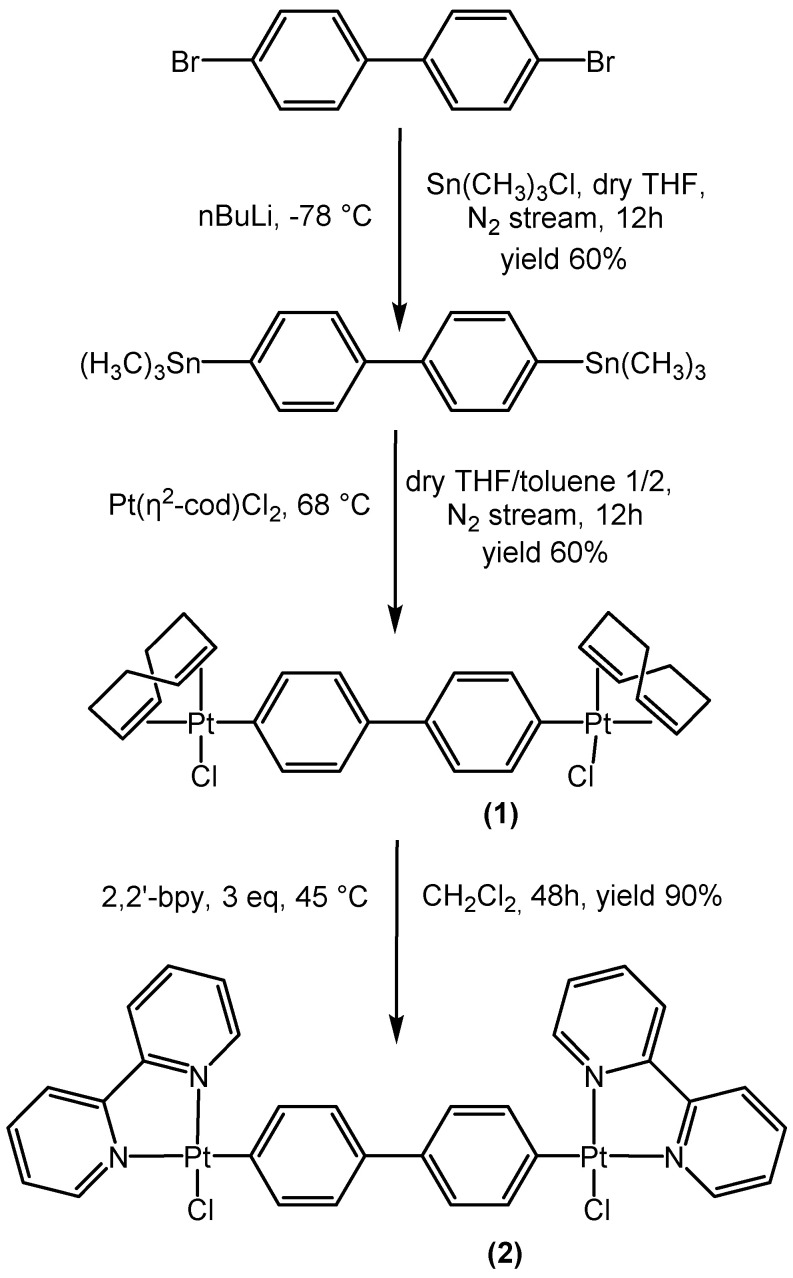
Reaction and conditions for the formation of complex (**2**).

Initially, the binuclear complex [Pt(*η*^2^-COD)Cl]_2_(*μ*-bph) (**1**) was synthesized through a slight modification of the method published by Yamago group [23]. The synthesis follows a three-step process as presented in Scheme 1. In the first step, a bromine–lithium exchange takes place at −78 °C in dry THF without isolating the reaction product. Then, a transmetallation reaction occurs between the 4,4′-lithiated-1,1′-biphenyl and (CH_3_)_3_SnCl, resulting in the formation of the arylstannane 4,4′-bis(trimethylstannyl)-1,1′-biphenyl in a good yield. Finally, a similar transmetallation reaction occurs between the arylstannane and the platinum complex Pt(*η*^2^-COD)Cl_2_, leading to the formation of the binuclear complex (**1**), which serves as the starting material for the subsequent reactions. The *η*^2^-coordinated ligand 1,5-cyclooctadiene (COD) in (**1**) was replaced by the chelating ligand 2,2′-bpy, in CH_2_Cl_2_, resulting in a nearly quantitative yield of the complex [Pt(bpy)Cl]_2_(*μ*-bph) (**2**). The addition of 2,2′-bpy to (**2**) caused a noticeable color change in the reaction mixture from colorless to bright yellow. 

**Figure 3:**
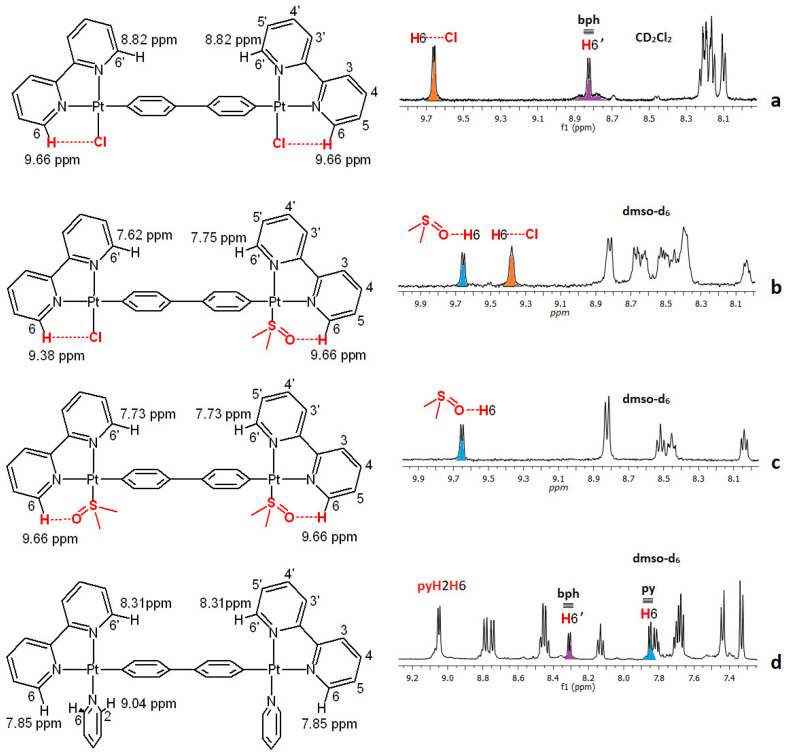
(**a**) Aromatic region of ^1^HNMR spectrum of complex (**2**) in CD_2_Cl_2_. (**b**) Aromatic region of ^1^HNMR spectrum of complex (**2**) in DMSO-*d*_6_. (**c**) Aromatic region of ^1^HNMR spectrum of complex (**3**) in DMSO-*d*_6_. (**d**) Aromatic region of ^1^HNMR spectrum of complex (**4**) in DMSO-*d*_6_.

In the ^1^H NMR spectrum of (**2**) in CD_2_Cl_2_, the half proton signals of the molecule were observed, reflecting its high symmetry. However, the two pyridine rings of 2,2′-bpy exhibit chemical nonequivalence, which can be attributed to a vertical orientation of bph ring system with respect to the plane of the 2,2′-bpy. This arrangement affects the chemical environment of the one pyridine ring of 2,2′-bpy that is positioned above the bph ring system, resulting in significant upfield shifts for its protons. Additionally, the proximity between the H6 of the other pyridine ring and the coordinated chlorine can result in interactions that further shift the H6 signal downfield. (Figure 3a). Additional evidence is present in the HR-ESI-MS of (**2**), where a cluster peak exists at *m*/*z* = 967.1224, assignable to the cation [C_34_H_31_N_4_ClSO^195^Pt_2_]^+^ (calc. *m*/*z* = 967.1224) which may formulated as {[Pt_2_(2,2′-bpy)_2_(DMSO)Cl](*μ*-bph)}^+^. The presence of the DMSO in the cation can be attributed to the addition of 5 μL DMSO to the sample in order to facilitate the solubility of (**2**). However, it seems that only partial replacement of the chloride takes place, as was also observed in the ^1^H NMR spectrum of (**2**) when it dissolved in dmso-*d*_6_ (Figure 3b). Thus, the symmetry of compound (**2**) was reduced, resulting in the appearance of two distinct sets of proton signals, one for each pyridine moiety of 2,2′-bpy. Under these circumstances, the H6 of 2,2′-bpy interacts with two different ligands: the coordinated chlorine located at one site of (**2**), which causes a downfield shift at 9.38 ppm, and the oxygen atom of the DMSO situated at the other site of (**2**), leading to a downfield shift at 9.66 ppm. This observation implies a stronger interaction between H6 and DMSO compared to that of chlorine (Figure 3a). The *syn*-conformation of the two chlorines in complex (**2**), which promotes the formation of the tetranuclear squares, has been observed in similar complexes as well [42].

Attempts to react directly complex (**2**) with pyridinic ligands were unsuccessful due to its extremely low solubility in many organic solvents. Thus, the replacement of chlorines was achieved by adding an equimolar amount of AgNO_3_ in MeCN and subjecting the mixture to sonication using a 750 W sonicator. The ^1^HNMR spectrum of {[Pt(2,2′-bpy)(MeCN)]_2_(*μ*-bph)}(NO_3_)_2_ in DMSO-*d*_6_ indicates that the pyridine rings of 2,2′-bpy remain nonequivalent due to the vertical orientation of bph ring system towards the plane of the 2,2′-bpy. Furthermore, the replacement of the coordinated MeCN by DMSO-*d*_6_ influences the chemical shift of 2,2′-bpyH6, which is observed at 9.66 ppm. This finding supports the hypothesis of a mixed Cl- DMSO adduct when (**2**) is dissolved in DMSO-*d*_6_. (Figure 3b) In order to further investigate the impact of the coordinated ligand on 2,2′-bpyH6, we synthesized the complex [Pt(2,2′-bpy)(py)]_2_(*μ*-bph), wherein the two MeCN ligands were replaced by pyridines. In the ^1^H NMR spectrum of (**4**), the H6 signal appears at 7.85 ppm, shifted significantly upfield compared to (**2**) and (**3**), suggesting a strong shielding effect from the pyridine ring. (Figure 3c). The assignment was assisted using the ROESY spectrum of (**4**), where a distinct ROE cross-peak between the pyH6H2 and 2,2′-bpyH6 protons was observed (Figure S1).

**Figure 4:**
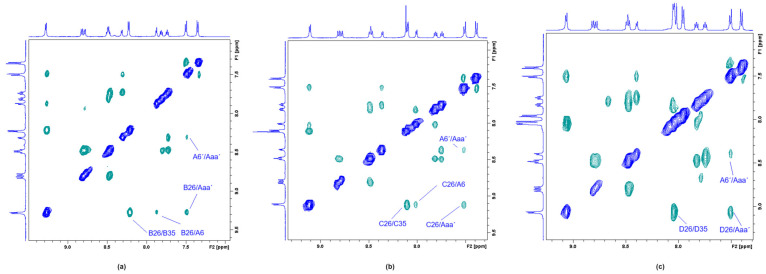
Aromatic region of ^1^H-^1^H ROESY spectrum of complex (**8**) (**a**), (**9**) (**b**) and (**10**) (**c**) in DMSO-*d*_6_ at 298 K in 500 MHz with assignment at interligand cross-peaks.

**Scheme 2:**
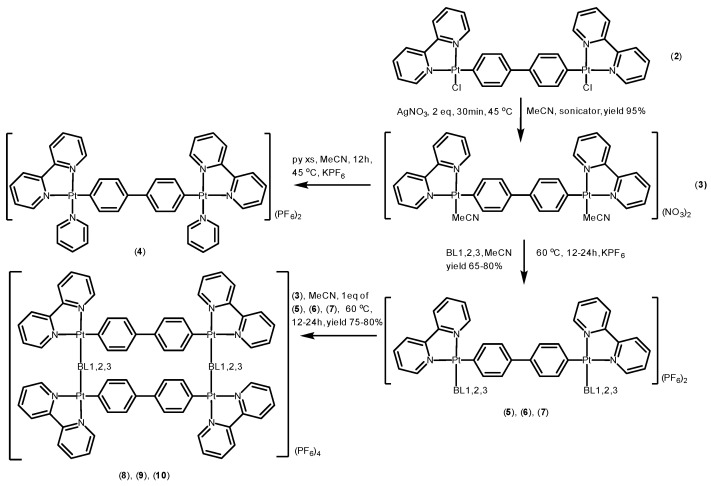
Synthetic procedure of formation of complexes (**8**)–(**10**).

Subsequently, complex (**3**) reacted with an excess of 4,4′-bpy (BL-1), resulting in the formation of {[Pt(2,2′-bpy)(4,4′-bpy)]_2_(*μ*-bph)}{PF_6_}_2_, (**5**). The monodentate coordination of 4,4′-bpy was evident from the nonequivalence of its pyridine rings. Additionally, we observed a coordination-induced-shift of 4,4′-bpyH6 of the coordinated pyridine ring by +0.48 ppm, while the non-coordinating pyridine ring showed only a slight shift of +0.06 ppm. The significant deshielding effect on 2,2′-bpyH6 (Δδ = −1.68 ppm) due to its proximity to the pyridine ring of 4,4′-bpy is analogous to that observed in the case of (**4**). Similarly, complex (**3**) was treated with BL-2 and BL-3, leading to the formation of the pi-shaped complexes (**6**) and (**7**). The tetranuclear metallocycles (**8**), (**9**) and (**10**) were synthesized by reacting equimolar amounts of the pi-shaped complexes (**5**), (**6**) and (**7**) with complex (**3**) in MeCN (yields 75–80%). Their ^1^H NMR spectra are simplified compared to their respective initial pi-shaped complexes, since the BL is now symmetrically coordinated. In the ROESY spectra, strong cross-peaks between 2,2′-bpyH6/BLH6, 2,2′-bpyH6′/bphHa and 4,4′-bpyH6/bphHa were observed (Figure 4), indicating that both the BL and bph aromatic ring planes are perpendicular to the plane of 2,2′-bpy. This structural feature could provide valuable insights into their intermolecular interactions. The synthetic route described above is summarized in Scheme 2.

**Figure 5:**
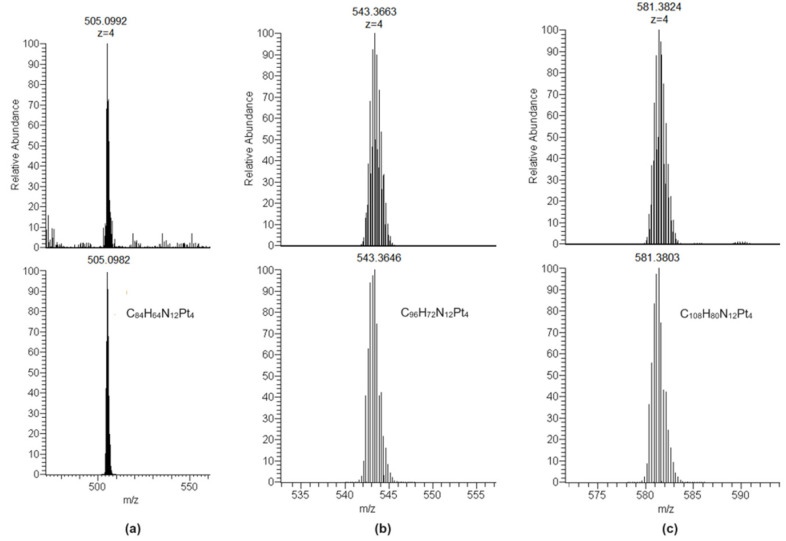
(**a**) HR-ESI MS spectra of complex (**8**) for the cation {M-4[PF_6_]}^4+^. (**b**) HR-ESI MS spectra of complex (**9**) for the cation {M-4[PF_6_]}^4+^. (**c**) HR-ESI MS spectra of complex (**10**) for the cation {M-4[PF_6_]}^4+^.

The formation of the SCCs (**8**)–(**10**) was further confirmed through high-resolution ESI-MS analysis. In all spectra, three cluster peaks were observed, assignable to the multicharged cations generated after the successive release of the [PF_6_]^−^ from the original complex. Therefore, the following cations were assigned: {M-4[PF_6_]}^4+^, {M-3[PF_6_]}^3+^ and {M-3[PF_6_]}^2+^, with the {M-4[PF_6_]}^4+^ being the most abundant among them. The isotopic patterns of these cations were found to match the simulated theoretical ones, as illustrated in Figure 5.

### 2.2. Crystal Structure of Complex ***8***

**Figure 6:**
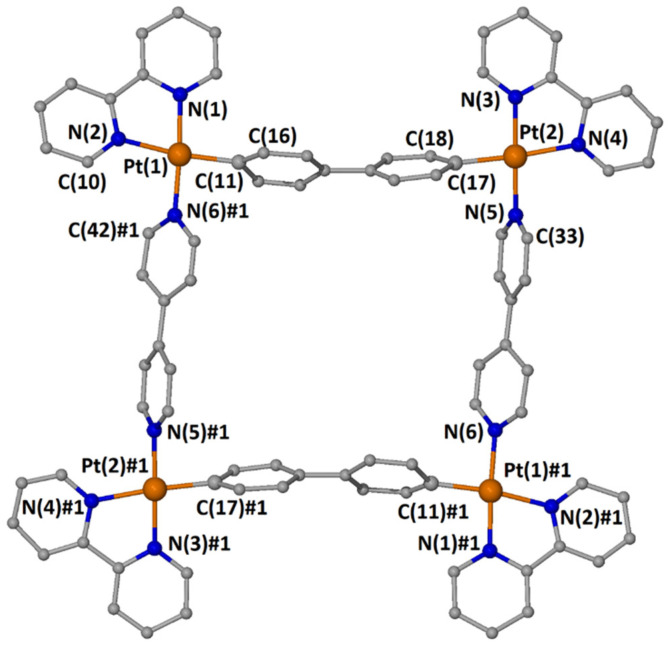
A partially labeled ball-and-stick diagram of the cation’s structure in **8**. Hydrogen atoms have been omitted for clarity. Symmetry transformation used to generate equivalent atoms: #1, −x, −y + 2, −z + 1.

Compound **8**, formulated as {[Pt(2,2′-bpy)]_4_(*μ*-bph)_2_(*μ*-(4,4′-bpy)_2_}{PF_6_}_4_, crystalizes in the monoclinic space group *P*$\bar{1}$. The cation is presented in Figure 6, while selected bond distances and angles are presented in Table 1. The complex is tetranuclear and its asymmetric unit consists of two platinum atoms bridged by a bph^2−^ moiety, a 4,4′-bpy molecule bonded to Pt(2) and two chelated 2,2′-bpy molecules. The cation’s charge is balanced by two PF_6_^−^ anions, with one of them being severely disordered.

As expected for d^8^ Pt(II) complexes, the Pt atoms in the two symmetry independent coordination sites are four-coordinate, showing distorted square planar geometries. The distortions are attributed to the bite angle of the chelated 2,2′-bpy. Both the coordination sphere and the Pt(2,2′-bpy) units are essentially planar, while the Pt–N and Pt–C bond lengths and angles in the two PtCN_3_ moieties are similar and close to those observed in similar complexes such as [Pt_4_(4,4′-bpy)_4_(LH)_4_](PF_6_)_4_, where LH_2_ is 2,6-diphenylpyridine [22] and [Pt(2,2′-bpy)L](PF_6_), where LH is 2-(4-(ethoxycarbonyl)quinolin-2-yl)benzene [43]. The Pt–N bonds *trans* to Pt–C bonds (mean value 2.102 Å) are slightly but systematically longer than the other Pt–N bonds (mean value 1.987 Å), emphasizing the *trans* influence of the coordinated carbon atom on Pt(II).

A common characteristic of the two coordination sites is the twisted orientation of the 4,4′-bpy and bph rings with respect to the plane of the Pt(2,2′-bpy) units. The corresponding dihedral angles are: N(1)–Pt(1)–C(11)–C(16), 77.3; N(2)–Pt(1)–N(6)#1–C(42)#1, 54.0; N(3)–Pt(2)–C(17)–C(18), 83.3; N(4)–Pt(2)–N(5)–C(33), 73.5°. It is likely that this orientation of the aromatic ligand rings is adopted to relieve the steric repulsion between the hydrogen atoms bonded on the *ortho* positions of the coordinated atoms.

An accurate description of the arrangement formed by the four platinum atoms is a parallelogram, since it is planar due to symmetry, the angles deviate significantly from 90°, and the two diagonals are different, as shown in Table 1.

The lattice constituents interact with each other with a variety of supramolecular interactions involving π–π stacking between the 2,2′-bpy rings and non-conventional C–H⋯F H-bonds. There is a significant amount of void space in the structure (approximately 13.7% of the unit cell volume) which hosted some electron density that belongs probably to the crystallization solvents and could not be modeled. 

### 2.3. Photophysical Studies

#### 2.3.1. Absorption and Emission Spectra of (**8**)–(**10**)

**Figure 7:**
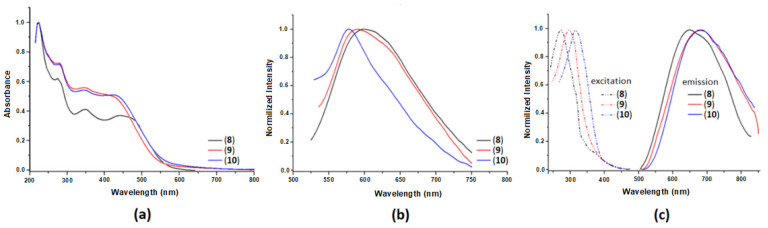
(**a**) Solid state absorption spectra of (**8**)–(**10**). (**b**) Solid state emission spectra at room temperature of (**8**)–(**10**). (**c**) Excitation and emission spectra of (**8**)–(**10**) in CH_3_CN (5 × 10^−6^ M).

Absorption and emission spectral data of the complexes (**8**)–(**10**) are summarized in Table 2. Figure 7a,b display the solid-state UV-vis absorption and emission spectra of (**8**)–(**10**). 

The solid-state absorption spectra of (**8**)–(**10**) exhibit similar features, with the absorption bands observed at 200–350 nm attributed to intraligand transitions of the C^C bph and N,N′ of 4,4-bpy, dpbz and dpbph. The lowest absorption bands at 442 (**8**), 425 nm (**9**), and 423 (sh) (**10**) are assigned to metal-to-ligand charge transfer [(5d)Pt→π*(L)] transitions [44]. These absorption maxima follow the order (**8**) > (**9**) ≈ (**10**), and blue-shifted as the length of the BL increases, which can be attributed to the reduced CT efficiency caused by the increased length of the BL [45]. 

In acetonitrile solutions, the UV-vis spectra of the complexes (**8**)–(**10**) (Figure 7c) exhibit a broad band spanning the range of 250 to 400 nm, with their λ_max_ values centered at 273, 295 and 315 nm, which can be assigned to intraligand transitions. Notably, the spectra’s maxima demonstrate that shifts are proportional to the expansion of N,N′ ligand rings. This is particularly evident when introducing a phenyl ring, resulting in the anticipated red-shift [46]. The molar absorbance coefficients for these complexes were calculated to fall within the range of 17 to 12 × 10^4^ M^−1^cm^−1^, similar to those reported by Stang et al. for rhomboidal complexes featuring phenanthrene ligands [37]. Complexes previously mentioned in the literature generally exhibit similar behavior, with their absorbance plateau extending up to 400 nm [37,39,40,47]. However, a notable exception is found in a rhomboidal platinum complex with two -NH2 groups, substituted in 2,6-bis(4-pyridylethynyl)aniline, which displays an absorption band with λ_max_ at 480 nm [37].

Upon excitation at 400 nm, (**8**)–(**10**) emit orange-red light with λ_em_ centered at 602 (**8**), 581 (**9**) and 578 nm (**10**). The calculated Φ values range from 1.2 to 6.0. Again, a blue-shift is observed upon increasing the BL length. In diluted acetonitrile solutions, the emission maxima of (**8**)–(**10**) are observed at 646 (**8**), 667 (**9**) and 685 nm (**10**) with low Φ values (0.04–0.14%) (Figure 7c). The emission of complexes (**8**) and (**9**) is considerably red-shifted compared to most complexes mentioned in the literature, such as rhomboidal Pt(II) complexes with 2,6-bis(4-pyridylethynyl)aniline and anthracene, where λ_em_ was found at approximately 400 and 500 nm accompanied by moderate Φ values [39]. Among these, only the complex which has two -NH_2_ groups, substituted in the ligand, and mentioned previously, exhibits an emission band centered at 581 nm and quantum yield smaller than 1%. A similar phenomenon is observed in parallelogram complexes with 1,4-bis(4-pyridylethynyl)benzene and p-Phtalic acid, where emission bands are centered at 390 nm [40]. The excitation of samples at 400 nm produces an emission at approximately 600 nm, with a low quantum yield for complexes 9 and 10 (1%) and a moderate quantum yield for complex (**11**) (6%). In this case, a blue-shifting is observed due to the increase in aromaticity. The λ_max_ are red-shifted by 50–100 nm compared to those in the solid state, indicating that the emission in the solid state is influenced by aggregation-induced phenomena possibly caused either by Pt⋯Pt interactions or/and by intermolecular ligand stacking [48], enhancing the quantum efficiency as well. However, contrary to the observation in the solid state, the emission λ_max_ of (**8**)–(**10**) in acetonitrile undergoes a red-shift upon increasing the BL length.

Transient photoluminescence measurements were carried out at 25 °C and λ_exc_ = 325 nm, and the results are summarized at Table 3. Complexes (**8**)–(**10**) have *τ* values that are about 10–100 times larger than parallelogram and rhomboidal ones reported in the literature [40,47]; however, they are within the normal range for organometallic compounds (10–700 ns) [49]. 

#### 2.3.2. Solvent Effect on Emission Spectra of (**8**)–(**10**) 

**Figure 8:**
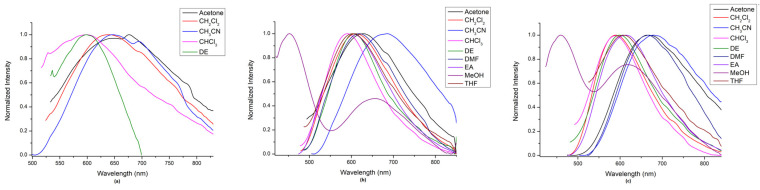
Emission spectra of complexes (**8**) (**a**), (**9**) (**b**) and (**10**) (**c**) in different polarity solvents (λ_exc._ = 365 nm).

The emission spectra of (**8**)–(**10**) were recorded in solvents with varying polarities to investigate their fluorosolvatochromic properties (Figure 8). The excitation wavelength λ_exc._ was kept constant at 365 nm for all cases. Complex (**8**) appears to be non-emissive in certain polar solvents such as DMF, EA, MeOH and THF. On the other hand, complexes (**9**) and (**10**) are emissive in all nine tested solvents, including methanol, wherein a bimodal emission pattern was observed. The emission band is located at orange region (590–614 nm) in slightly polar solvents, and red-shifted (620–670 nm) at highly polar solvents like methanol, acetone, acetonitrile and DMF. The results are presented in Table 4. Furthermore, in the spectrum of (**10**), the strongest emission is observed in CH_2_Cl_2_ compared with the other solvents (Φ = 4%), similar to (**8**) and (**9**) in the same solvent. The red-shift of the λ_em_ in highly polar solvents suggests that their excited states are more polarized than their ground states [41].

#### 2.3.3. Aggregation Effect and Emission Bimodal Spectra of (**9**) and (**10**) 

As previously mentioned, (Section 2.3.2) complexes (**9**) and (**10**) exhibit dual-emission spectra in MeOH, emitting at λ_em_ 455/659 nm and 461/630 nm, respectively. Dual emitters have attracted considerable attention for various applications, such as fluorescent sensors [51], bioimaging [52], and single-molecule white light-emitting diodes [53] among others. For the latter, the tuning of the emission color may achieved by combining the λ_em_ of a dual emitter with appropriate portions in different matrices [54]. Aggregation-induced emission (AIE) and aggregation-caused quenching (ACQ) are opposing phenomena [55]. AIE refers to luminogenic compounds that are non-emissive when they are well dissolved in solvents as isolated molecules but become highly luminescent when they are aggregated in solvents with low solubility. AIE is the result of rapid energy dissipation by crossing a conical intersection in solutions, leading to low luminescence efficiencies and, in solids, intermolecular coupling, which effectively transfers energy and prevents quenching [56]. On the other hand, ACQ refers toa reduction in emission intensity due to the aggregation of molecules into nanostructures, resulting in a smaller energy gap between the first singlet-excited state and the first triplet-excited state [57]. 

**Figure 9:**
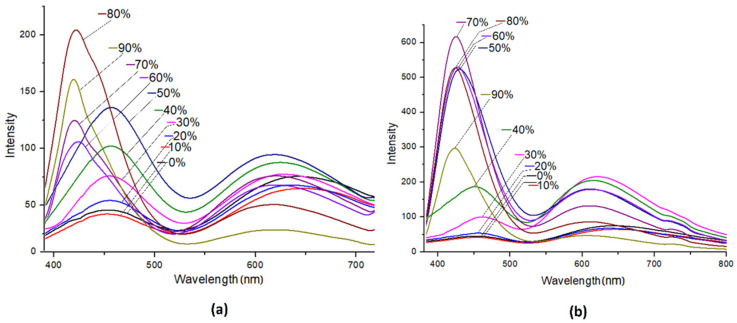
(**a**) Emission spectrum of complex (**9**) increasing in a mixture of MeOH/H_2_O in different ratios. (**b**) Emission spectrum of complex (**10**) increasing in a mixture MeOH/H_2_O in different ratios.

In order to investigate the influence of aggregation in dual emissions of the complexes (**8**) and (**9**), titrations of the complexes in methanol with a solvent capable of forming molecular aggregates, such as water, was carried out (Figure 9).

In the case of complex (**9**), as the percentage of H_2_O increases up to 50%, the intensity of the peak at 659 nm also increases, as well as the peak at 455 nm, which slightly blue-shifted (Δν = 40 nm). This could be attributed to the molecular aggregation of (**9**) due to hydrophobic interactions in a 1:1 mixture of water and methanol. Similar results have been reported by Fan et al., wherein the induced emission of a dual-emitter rhomboidal metallacycle in a solvent mixture of MeCN/hexane was attributed to the formation of aggregates [41]. However, when the H_2_O percentage exceeds 50%, a sharp change in the spectrum is observed, followed by a significant blue-shift in the λ_em_ emission (Δν = 35 nm) and an increase in intensity. Simultaneously, the intensity of the orange emission significantly decreases. This sharp change may be attributed to the transition from the aggregate phase to the solid phase, through the precipitation of the complex in such a mixture of solvents. Similarly, in the case of complex (**10**), with increasing the percentage of H_2_O up to 40%, the intensity of the peak at 630 nm increases, and a blue-shift of Δν = 15 nm is observed, while the intensity of the peak at 461 nm increases but remains almost at the same λ_em_. This similarity with complex (**9**) suggests that the origin of the phenomenon is likely the same, originating from the molecular aggregation. The slight differences that appear are probably due to the differences in their size and solubility in MeOH/H_2_O, as the precipitation starts at relatively lower ratios MeOH:H_2_O [1:1 for (**9**) and 1:1.5 for (**10**)]. When the H_2_O percentage exceeds 40%, a smoother change than in the case of (**9**) is observed. This is followed by a blue-shift of the λ_em_ emission (Δν = 15 nm) and a significant increase in intensity. Simultaneously, the intensity of the orange emission significantly decreases, reaching almost zero in 90% water, which may be attributed to the nearly complete precipitation of (**10**).

## 3. Experiment

### 3.1. Materials and Methods

All solvents were of analytical grade and were used without further purification, except for tetrahydrofuran, which was distilled from sodium benzophenone ketyl and toluene, which was distilled from CaH_2_ and stored over molecular sieves. Briefly, 2,2′-bipyridine, 4,4′-bipyridine and 1,4-di(pyridin-4-yl)benzene were purchased from Fluorochem, 4,4′-di(pyridin-4-yl)-1,1′-biphenyl was purchased from TCI, and Pt(COD)Cl_2_, Sn(CH_3_)_3_Cl, *n*-BuLi (2,5M in hexane) and 4,4′-dibromo-1,1′-biphenyl were purchased from Sigma. Moreover, 4,4′-bis(trimethylstannyl)-1,1′-biphenyl and the complex [Pt(*η*^2^-COD)Cl]_2_(*μ*-bph) were prepared according to methods from the literature [23]. 

^1^H NMR spectra of the ligands and the complexes were recorded using a Bruker Avance II spectrometer operating at a ^1^H frequency of 500.13 MHz or 400.13 MHz, and were processed using Topspin 4.2 (Bruker Analytik GmbH, Bremen, Germany). Two-dimensional COSY, TOCSY, and ROESY spectra were recorded following standard Bruker procedures. High-resolution electrospray ionization mass spectra (HR-ESI-MS) were obtained using a Thermo Scientific LTQ Orbitrap XL™ system. The UV–vis spectra of the complexes were recorded on an Agilent Cary 60 UV–vis spectrophotometer with a xenon source lamp in MeCN at room temperature. The fluorescence lifetime of complexes (**8**)–(**10**) was measured using an Edinburgh Mini-tau Lifetime Spectrometer. The sonication of the reaction between [Pt(bpy)Cl]_2_(*μ*-bph) and AgNO_3_ in MeCN was achieved using a Sonics and Materials instrument. 

### 3.2. Fluorescence Emission Studies

Emission studies were carried out using a Jasco FP-8300 fluorometer equipped with a xenon lamp source and an integrated sphere for solid samples. The relative quantum yield for solutions was determined using the equation Qs = Q_r_(A_r_/A_s_)(E_s_/E_r_)(n_s_/n_r_)², with a water solution of [Ru(bpy)₃]Cl₂ serving as the reference standard (Q_r_ = 0.04). In this equation, ‘A’ represents the absorbance of the solutions, ‘E’ stands for the integrated fluorescence intensity of the emission spectrum, and ‘n’ denotes the refractive index of the solvents. Subscripts ‘r’ and ‘s’ correspond to the reference and sample, respectively. The relative quantum yield of the solid-state complexes was calculated using the following equation: Q = S₂/(S₀ − S₁), where ‘Q’ represents the quantum yield of the solid state of the complexes. In this equation, ‘S₂’ denotes the integrated emission intensity of the sample, while ‘S₀’ and ‘S₁’ refer to the excitation intensities of the standard and the sample, respectively.

### 3.3. X-ray Crystallography 

Crystals from compound **8** were grown via slow vapor diffusion of diethyl ether in an acetonitrile solution of the prepared complex.

A suitable crystal of approximate dimensions 0.02 × 0.06 × 0.12 mm^3^ was glued to a thin glass fiber with cyanoacrylate (super glue) adhesive and placed on the goniometer head. Diffraction data were collected on a Bruker D8 Quest Eco diffractometer, equipped with a Photon II detector and a TRIUMPH (curved graphite) monochromator utilizing Mo Kα radiation (λ = 0.71073 Å) using the APEX3 software package [58]. The collected frames were integrated with the Bruker SAINT software using a wide-frame algorithm. Data were corrected for absorption effects using the multi-scan method (SADABS) [59]. The structure was solved using the Bruker SHELXT Software Package and refined via full-matrix least-squares techniques on F2 (SHELXL 2018/3) [60] via the ShelXle interface [61]. The non-H atoms were treated anisotropically, while the organic H atoms were placed in calculated, ideal positions and refined as riding on their respective carbon atoms. 

Details on data collection and refinement are presented in Table S1. Full details on the structures can be found in the CIF files deposited with CCDC. CCDC 2296015 contains the supplementary crystallographic data for this paper.

### 3.4. Synthesis of the Complexes *(**2**)–(**10**)*

[Pt(2,2′-bpy)Cl]_2_(*μ*-bph), (**2**): 121.9 mg of 2,2′-bipyridine (0.78 mmol) was added to a suspension of 120 mL CH_2_Cl_2_ containing 97 mg (0.13 mmol) [Pt(*η*^2^-COD)Cl]_2_(*μ*-bph). The suspension was stirred for about 48 h at room temperature. After 48 h, the suspension turned into a light-yellow solution. The solvent was evaporated and the solid was washed with hexane and diethyl ether. Yield: 95%. ^1^H NMR: (500 MHz, 298 K, CD_2_Cl_2_, δ in ppm): AH6: 9.66 (d, 2H, ^3^J = 5.5); AH33′: 8.20 (d, 4H, ^3^J = 8.1); AH4: 8.11 (t, 2H, ^3^J = 8.0); AH4′: 7.78 (t, 2H, ^3^J = 8.2); AH5: 8.05 (t, 2H, ^3^J = 7.7); AH6′: 8.83 (d, 2H, ^3^J = 5.4); AH5′: 7.42 (t, 2H, ^3^J = 7.8); AHaa′: 7.49 (d, 4H, ^3^J = 8.4); AHbb′: 7.43 (d, 4H, ^3^J = 8.3). HR-ESI-MS (5 mL CH_2_Cl_2_ + 20 μL of DMSO), positive (*m*/*z*): found 967.1224, calc. 967.1176 for [C_34_H_31_N_4_ClSO^195^Pt_2_]^+^, assignable to the cation {[Pt_2_(2,2′-bpy)_2_(DMSO)Cl](*μ*-bph)}^+^. 

{[Pt(2,2′-bpy)(MeCN)]_2_(*μ*-bph)}(NO_3_)_2_, (**3**): 29.4 mg of AgNO_3_ (0.17 mmol) was added to a suspension containing 80 mg (0.086 mmol) of (**2**) in 12 mL MeCN. The mixture was sonicated for 30 min in 375 W (1/2″ probe) at 55 °C. After sonication, the mixture was filtered, and the solvent was evaporated. Yield 99%. ^1^H NMR: (500 MHz, 298 K, DMSO-*d*_6_, δ in ppm). AH6: 9.66 (d, 2H, ^3^J = 5.6); AH33′: 8.83 (d, 4H, ^3^J = 8.1); AH4: 8.52 (t, 2H, ^3^J = 8.0); AH4′: 8.46 (t, 2H, ^3^J = 8.0); AH5: 8.05 (t, 2H, ^3^J = 7.8); AH6′: 7.73 (d, 2H, ^3^J = 5.7); AH5′: 7.72 (t, 2H, ^3^J = 7.7); AHaa′: 7.64 (d, 4H, ^3^J = 8.3); AHbb′: 7.60 (d, 4H, ^3^J = 8.3). HR-ESI-MS (MeCN), positive (*m*/*z*): found 468.5961, calc. 468.5953 for [C_36_H_31_N_6_^195^Pt_2_]^2+^, assignable to the cation {[Pt(2,2′-bpy)(MeCN)]_2_(*μ*-bph)}^2+^.

{[Pt(2,2′-bpy)(py)]_2_(*μ*-bph)}{PF_6_}_2_, (**4**): 380 μL (0.42 mmol) of pyridine was added in 20 mL acetonitrile containing 25 mg (0.021 mmol) of (**3**) {[Pt(2,2-bpy)(MeCN)](*μ*-bph)}{NO_3_}_2_ and 9.8 mg (0.053) mmol of KPF_6._ The mixture was stirred for 12 h at room temperature. After the evaporation of solvent, the orange solid was washed with H_2_O several times. Yield 92%. ^1^H NMR: (500 MHz, 298 K, DMSO-*d*_6_, δ in ppm): pyH26: 9.05 (d, 4H, ^3^J = 5.1); AH3′: 8.79 (d, 2H, ^3^J = 8.1); AH3: 8.75 (d, 2H, ^3^J = 8.0); AH44′: 8.46 (t, 4H, ^3^J = 8.0); AH6′: 8.31 (d, 2H, ^3^J = 5.7); AH5′: 8.13 (t, 2H, ^3^J = 7.8); AH6: 7.85 (d, 2H, ^3^J = 5.7); pyH53: 7.71 (t, 4H, ^3^J = 5.7); AH5′: 7.70 (t, 2H, ^3^J = 7.7); pyH4: 7.68 (t, 2H, ^3^J = 7.8); AHaa′: 7.43 (d, 4H, ^3^J = 8.3); AHbb′: 7.33 (d, 4H, ^3^J = 8.3). HR-ESI-MS (MeCN), positive (*m*/*z*): found 468.5961, calc. 468.5953 for [C_36_H_31_N_6_^195^Pt_2_]^2+^, assignable to the cation {[Pt(2,2′-bpy)(MeCN)]_2_(*μ*-bph)}^2+^.

{[Pt(2,2′-bpy)(4,4′-bpy)]_2_(*μ*-bph)}{PF_6_}_2_, (**5**): 40.6 mg (0.26 mmol) of 4,4′-bpy was added to 40 mL acetonitrile containing 30 mg (0.026 mmol) of (**3**) {[Pt(2,2-bpy)(MeCN)](*μ*-bphen)}{NO_3_}_2_ and 9.8 mg (0.053) mmol of KPF_6_. The mixture was stirred for 18 h at room temperature. After the evaporation of solvent, the orange solid was washed with H_2_O and diethyl ether. Yield 80%. ^1^H NMR: (500 MHz, 298 K, DMSO-*d*_6_, δ in ppm): BH26: 9.18 (d, 4H, ^3^J = 6.7); BH2′6′: 8.78 (d, 4H, ^3^J = 6.5); AH3′: 8.76 (d, 2H, ^3^J = 8.1); AH3: 8.73 (d, 2H, ^3^J = 8.2); AH44′: 8.45 (t, 4H, ^3^J = 8.0); AH6′: 8.31 (d, 2H, ^3^J = 6.1); BH35: 8.12 (d, 4H, ^3^J = 6.7); AH6: 7.98 (d, 2H, ^3^J = 6.5); BH3′5′: 7.93 (d, 4H, ^3^J = 6.4); AH5: 7.80 (t, 2H, ^3^J = 6.7); AH5′: 7.70 (t, 2H, ^3^J = 6.9); AHaa′: 7.48 (d, 4H, ^3^J = 8.2); AHbb′: 7.35 (d, 4H, ^3^J = 8.2). HR-ESI-MS (MeCN), positive (*m*/*z*): found 468.5961, calc. 468.5953 for [C_36_H_31_N_6_^195^Pt_2_]^2+^, assignable to the cation {[Pt(2,2′-bpy)(MeCN)]_2_(*μ*-bph)}^2+^.

{[Pt(2,2′-bpy)(dpbz)]_2_(*μ*-bph)}{PF_6_}_2_, (**6**): 69.7 mg (0.30 mmol) of dpbz was added to 40 mL acetonitrile containing 35 mg (0.03 mmol) of (**3**) {[Pt(2,2-bpy)(MeCN)](*μ*-bphen)}{NO_3_}_2_ and 5.6 mg (0.053) mmol of KPF_6._ The mixture was stirred for 18 h at room temperature. After the evaporation of solvent, the orange solid was washed with acetone and diethyl ether. Yield 68%. ^1^H NMR: (500 MHz, 298 K, DMSO-*d*_6_, δ in ppm): CH26: 9.13 (d, 4H, ^3^J = 6.8); CH2′6′: 8.79 (d, 4H, ^3^J = 6.2); AH3′: 8.77 (d, 2H, ^3^J = 7.9); AH3: 8.70 (d, 2H, ^3^J = 8.5); AH44′: 8.48 (t, 4H, ^3^J = 8.2); AH6′: 8.32 (d, 2H, ^3^J = 5.9); CH35: 8.16 (d, 4H, ^3^J = 6.6); CHaa′bb′: 8.09 (s,8H); AH6: 8.04 (d, 2H, ^3^J = 6.5); CH3′5: 7.82 (d, 4H, ^3^J = 6.4); AH5: 7.82 (t, 2H, ^3^J = 6.9); AH5′: 7.74 (t, 2H, ^3^J = 7.2); AHaa′: 7.52 (d, 4H, ^3^J =7.9); AHbb′: 7.42 (d, 4H, ^3^J = 7.7).

{[Pt(2,2′-bpy)(dpbph)]_2_(*μ*-bph)}{PF_6_}_2_, (**7**): 112.2 mg (0.36 mmol) of dpbz was added to 70 mL acetonitrile containing 42 mg (0.036 mmol) of (**3**) {[Pt(2,2-bpy)(MeCN)](*μ*-bphen)}{NO_3_}_2_ and 13.3 mg (0.053) mmol of KPF_6._ The mixture was stirred for 24 h at room temperature. After the evaporation of solvent, the orange solid was washed with THF and diethyl ether. Yield 65%. ^1^H NMR: (500 MHz, 298 K, DMSO-*d*_6_, δ in ppm): DH26: 9.10 (d, 4H, ^3^J = 7.0); DH2′6′: 8.79 (d, 4H, ^3^J = 6.8); AH3′: 8.78 (d, 2H, ^3^J = 8.0); AH3: 8.76 (d, 2H, ^3^J = 8.1); AH44′: 8.47 (t, 4H, ^3^J = 7.7); AH6′: 8.31 (d, 2H, ^3^J = 6.1); DH35: 8.11 (d, 4H, ^3^J = 6.7); AH6: 8.09 (d, 2H, ^3^J = 6.9); DHaa′bb′: 8.99 (s, 8H); DH3′5′: 7.99 (d, 4H, ^3^J = 6.4); AH5: 7.80 (t, 2H, ^3^J = 6.7); AH5′: 7.74 (t, 2H, ^3^J = 7.0); AHaa′: 7.52 (d, 4H, ^3^J = 8.2); AHbb′: 7.41 (d, 4H, ^3^J = 8.2).

The tetranuclear Pt(II) complexes (**8**)–(**10**) were synthesized similarly. In a typical experiment, 1 eq. of the bridging ligands (4,4′-bpy, dpbz, dpbph) was added in 20–30 mL acetonitrile containing 1 eq. of the binuclear (**5**)–(**7**)_._ The mixture was heated for 12–18 h at 60 °C. After 19 h, the reaction mixture was evaporated to dryness under reduced pressure, and the orange solid was washed several times with DCM and diethyl ether.

{[Pt(2,2′-bpy)]_4_(*μ*-bph)_2_(*μ*-(4,4′-bpy)_2_}{PF_6_}_4_, (**8**): 3.1 mg (0.019 mmol) of 4,4′-bpy was added to 20 mL acetonitrile containing 30 mg (0.019 mmol) of complex **5**. Yield: 75%. ^1^HNMR: (500 MHz, 298 K, DMSO-*d*_6_, δ in ppm): ΒH26: 9.28 (d, 8H, ^3^J = 6.6); AH3′: 8.82 (d, 4H, ^3^J = 8.4); AH3: 8.78 (d, 4H, ^3^J = 8.6); AH44′: 8.48 (tt, 8H, ^3^J = 7.0); AH6′: 8.31 (d, 4H, ^3^J = 5.2); BH35: 8.22 (d, 8H, ^3^J = 6.9); AH6: 7.87 (d, 4H, ^3^J = 5.0); AH5: 7.80 (t, 4H, ^3^J = 6.88); AH5′: 7.73 (t, 4H, ^3^J = 6.10); AHaa′: 7.50 (d, 8H, ^3^J = 8.1); AHbb′: 7.35 (d, 8H, ^3^J = 8.3). HR-ESI-MS, positive (*m*/*z*): found 505.0996; calc. 505.0982 for [C_84_H_64_N_12_^195^Pt_4_]^4+^; found 722.1222; calc. 722.1217 for [C_84_H_64_N_12_PF_6_^195^Pt_4_]^3+^; found 1155.6649; calc. 1155.6627 for [C_84_H_64_N_12_P_2_F_12_^195^Pt_4_]^2+^.

{[Pt^II^(2,2′-bpy)]_4_(*μ*-bph)_2_(*μ*-dpbz)_2_}{PF_6_}_4_ (**9**): 6.2 mg (0.026 mmol) of dpbz was added to 20 mL acetonitrile containing 45 mg (0.026 mmol) of complex **6**_._ Yield: 68%. ^1^HNMR: (500 MHz, 298 K, DMSO-*d*_6_, δ in ppm): CH26: 9.11 (d, 8H, ^3^J = 6.8); AH3: 8.81 (d, 4H, ^3^J = 8.5); AH3′: 8.78 (d, 4H, ^3^J = 8.6); AH44′: 8.48 (tt, 8H, ^3^J = 7.8); AH6′: 8.36 (d, 4H, ^3^J = 5.1); CHab: 8.11 (s, 8H); AH5: 8.10 (d, 4H, ^3^J = 6.9); AH6: 8.00 (d, 8H, ^3^J = 7.2); AH5: 7.81 (t, 4H, ^3^J = 6.3); AH5′: 7.74 (t, 4H, ^3^J = 7.0); AHaa′: 7.51 (d, 8H, ^3^J = 8.1); AHbb′: 7.38 (d, 8H, ^3^J = 8.3). HR-ESI-MS, positive (*m*/*z*): found 543.3663; calc. 543.3646 for [C_96_H_72_N_12_^195^Pt_4_]^4+^; found 772.8104; calc. 772.8078 for [C_96_H_72_N_12_PF_6_^195^Pt_4_]^3+^; found 1231.6996; calc. 1231.6940 for [C_96_H_72_N_12_P_2_F_12_^195^Pt_4_]^2+^.

{[Pt^II^(2,2′-bpy)]_4_(*μ*-bph)_2_(*μ*-dpbph)_2_}{PF_6_}_4_ (**10**): 5.2 mg (0.017 mmol) of dpbph was added to 30 mL acetonitrile containing 31 mg (0.017 mmol) of complex (**7**)_._ Yield: 50%. ^1^HNMR: (500 MHz, 298 K, DMSO-*d*_6_, δ in ppm): DH26: 9.06 (d, 8H, ^3^J = 6.1); AH3: 8.80 (d, 4H, ^3^J = 8.4); AH3′: 8.77 (d, 4H, ^3^J = 8.6); AH44′: 8.47 (t, 4H, ^3^J = 7.4); AH6′: 8.39 (d, 4H, ^3^J = 5.5); AH6, DH35: 8.04 (dd, 12H, ^3^J = 5.9); DHbb′: 8.02 (dd, 8H, ^3^J = 7.4); DHaa′: 7.95 (d, 8H, ^3^J = 8.4); AH5: 7.83 (t, 4H, ^3^J = 6.1); AH5′: 7.74 (t, 4H, ^3^J = 6.8); AHaa′: 7.50 (d, 8H, ^3^J = 7.8); AHbb′: 7.39 (d, 8H, ^3^J = 8.2). HR-ESI-MS, positive (*m*/*z*): found 581.3824; calc. 581.3803 for [C_108_H_80_N_12_^195^Pt_4_]^4+^; found 823.4981; calc. 823.4953 for [C_108_H_80_N_12_PF_6_^195^Pt_4_]^3+^; found 1307.7301; calc. 1307.7253 for [C_108_H_80_N_12_P_2_F_12_^195^Pt_4_]^2+^.

## 4. Conclusions

Platinum(II) supramolecular coordination complexes (SCCs) (**8**)–(**10**) were synthesized using a stepwise method and fully characterized. The structural features of (**8**)–(**10**) indicate that they have a rectangular shape, especially complex (**8**), while complexes (**9**) and (**10**) can be referred to as parallelograms with four platinum atoms at the corners. This is also confirmed by the crystal structure of (**8**). The complexes’ emission spectra in solution showed a strong dependence on solvent polarity. Complexes (**9**) and (**10**) exhibited dual emission in MeOH, while (**8**) was non-emissive. Furthermore, (**9**) and (**10**) exhibited aggregation-induced emission (AIE) and quenching-induced emission (QIE) phenomena upon aggregation in MeOH/H_2_O solutions. 

## Figures and Tables

**Table 1:** Selected geometrical characteristics of the tetranuclear cation in (**8**).

Bond Lengths (Å)			
Pt(1)–N(1)	1.996(11)	Pt(1)–C(11)	1.971(18)
Pt(1)–N(2)	2.083(15)	Pt(2)–C(17)	1.99(2)
Pt(2)–N(3)	1.98(2)	Pt(2)–N(5)	1.966(11)
Pt(2)–N(4)	2.121(19)	Pt(1)–N(6)#1	2.009(17)
Pt(1)⋯Pt(2)	11.086(1)	Pt(1)⋯Pt(2)#1	11.050(1)
Pt(1)⋯Pt(1)#1	15.124(2)	Pt(2)⋯Pt(2)#1	16.162(2)
Bond angles (°)			
C(11)–Pt(1)–N(1)	96.5(8)	N(5)–Pt(2)–N(3)	178.3(7)
C(11)–Pt(1)–N(6)#1	87.4(8)	N(5)–Pt(2)–C(17)	84.9(7)
N(1)–Pt(1)–N(6)#1	173.2(6)	N(3)–Pt(2)–C(17)	96.0(9)
C(11)–Pt(1)–N(2)	172.4(8)	N(5)–Pt(2)–N(4)	100.0(7)
N(1)–Pt(1)–N(2)	77.3(7)	N(3)–Pt(2)–N(4)	79.1(9)
N(6)#1–Pt(1)–N(2)	99.2(7)	C(17)–Pt(2)–N(4)	174.4(8)
Pt(1)⋯Pt(2)⋯Pt(1)#1	86.20(1)	Pt(2)⋯Pt(1)⋯Pt(2)#1	93.80(2)

Symmetry transformations used to generate equivalent atoms: #1, −x, −y + 2, −z + 1.

**Table 2:** Photophysical data of complexes (**8**)–(**10**) (sh:shoulder).

Complex	UV/Vis Absorbance λ_max_ [nm], (ε × 10^4^ [M^−1^cm^−1^])		Excitation		Emission		QY%	
	Solution (MeCN)	Solid	λ_exc_		λ_em_			
			Solution (MeCN)	Solid	Solution (MeCN)	Solid	Solution (MeCN)	Solid
(**8**)	273 (17.90) 312sh (11.2) 335sh (9.18) 363sh (2.20)	225,275, 350, 440	365	400	646	602	0.04	1.1
(**9**)	295 (15.62)	225, 280, 345, 420	365	400	667	581 624 (sh)	0.08	1.2
(**10**)	315 (12.51)	225, 280, 345, 420	365	400	685	577	0.14	6.0

**Table 3:** Rate constants of complexes (**8**)–(**10**) in MeCN solution. kr = Φ*τ*^−1^, knr = *τ*^−1^ − kr [50].

	*τ* (10^−9^ s)		kr/(10^7^ s^−1^)		knr/(10^7^ s^−1^)	
	*τ*_1_	*τ*_2_	kr_1_	kr_1_	knr_1_	knr_2_
(**8**)	4.85	34.07	0.82	0.17	19.8	2.82
(**9**)	9.65	34.04	0.83	0.23	9.53	27.0
(**10**)	6.93	38.57	2.0	0.36	12.4	2.23

**Table 4:** λ_em_ and quantum yields (Φ) of complexes (**8**)–(**10**) in solvents with different polarities. DMF: N,N′-dimethylformamide, MeCN: acetonitrile, MeOH: methanol, EA: ethylacetate, THF tetrahydrofuran, DE: diethyl ether. (NE: non emissive).

Solvent	λ_em_ (Φ %)		
	(8)	(9)	(10)
DMF	NE	614 (0.01)	670 (0.22)
MeCN	646 (0.04)	667 (0.08)	685 (0.14)
MeOH	NE	659 (0.01) 455	630 (0.31) 461
Acetone	644 (0.02)	622 (0.01)	669 (0.58)
CHCl_3_	605 (0.01)	592 (0.01)	590 (0.13)
EA	NE	598 (0.01)	614 (0.48)
THF	NE	602 (0.01)	609 (0.02)
CH_2_Cl_2_	639 (0.01)	604 (0.04)	592 (4.09)
DE	612 (0.01)	598 (0.01)	601 (0.12)

## Data Availability

Not applicable.

## Supplementary Materials

The following supporting information can be downloaded at: https://www.mdpi.com/article/10.3390/molecules28217261/s1, Table S1: Crystal data and structure refinement for {[Pt(2,2′-bpy)]_4_(*μ*-bph)_2_(*μ*-(4,4′-bpy)_2_}{PF_6_}_4_ (**8**): details on the refinement procedure; Figure S1: Aromatic region of 1H-1H ROESY spectrum of complex (**4**) in DMSO-d_6_ at 298 K in 500 MHz with assignment at interligand cross-peaks; Figure S2: A packing diagram of compound (**8**) down to the axis of the unit cell. The void space is located within the parallelogram cations; Figure S3: Fluorescence lifetimes for (a) (**8**), (b) (**9**) and (c) (**10**). References [62,63] are cited in the supplementary materials.

## Sample Availability

Samples of the compounds (**8**)–(**10**) are available from the authors.

## References

[1] Saygili Y., Stojanovic M., Flores-Díaz N., Zakeeruddin S.M., Vlachopoulos N., Grätzel M., Hagfeldt A. (2019). Metal coordination complexes as redox mediators in regenerative dye-sensitized solar cells. Inorganics.

[2] Grifoni F., Bonomo M., Naim W., Barbero N., Alnasser T., Dzeba I., Giordano M., Tsaturyan A., Urbani M., Torres T. (2021). Toward Sustainable, Colorless, and Transparent Photovoltaics: State of the Art and Perspectives for the Development of Selective Near-Infrared Dye-Sensitized Solar Cells. Adv. Energy Mater..

[3] Bizzarri C., Spuling E., Knoll D.M., Volz D., Bräse S. (2018). Sustainable metal complexes for organic light-emitting diodes (OLEDs). Coord. Chem. Rev..

[4] Zhang Q.C., Xiao H., Zhang X., Xu L.J., Chen Z.N. (2019). Luminescent oligonuclear metal complexes and the use in organic light-emitting diodes. Coord. Chem. Rev..

[5] Zhang L., Humphrey M.G. (2022). Multiphoton absorption at metal alkynyl complexes. Coord. Chem. Rev..

[6] Colombo A., Dragonetti C., Guerchais V., Hierlinger C., Zysman-Colman E., Roberto D. (2020). A trip in the nonlinear optical properties of iridium complexes. Coord. Chem. Rev..

[7] Ning Y., Jin G.Q., Wang M.X., Gao S., Zhang J.L. (2022). Recent progress in metal-based molecular probes for optical bioimaging and biosensing. Curr. Opin. Chem. Biol..

[8] Lee L.C., Lo K.K. (2022). Strategic Design of Luminescent Rhenium(I), Ruthenium(II), and Iridium(III) Complexes as Activity-Based Probes for Bioimaging and Biosensing. Chem. Asian J..

[9] Dey N., Haynes C.J.E. (2021). Supramolecular Coordination Complexes as Optical Biosensors. Chempluschem.

[10] Fujita M., Yazaki J., Ogura K. (1990). Preparation of a macrocyclic polynuclear complex, [(en)Pd(4,4′-bpy)]4(NO3)8 (en = ethylenediamine, bpy = bipyridine), which recognizes an organic molecule in aqueous media. J. Am. Chem. Soc..

[11] Stang P.J., Whiteford J.A. (1994). Mixed, Neutral-Charged, Platinum-Platinum and Platinum-Palladium Macrocyclic Tetranuclear Complexes. Organometallics.

[12] Chakrabarty R., Mukherjee P.S., Stang P.J. (2011). Supramolecular Coordination: Self-Assembly of Finite Two- and Three-Dimensional Ensembles. Chem. Rev..

[13] Holliday B.J., Mirkin C.A. (2001). Strategies for the Construction of Supramolecular Compounds through Coordination Chemistry. Angew. Chem. Int. Ed..

[14] Fujita M., Yazaki J., Ogura K. (1991). Spectroscopic Observation of Self-Assembly of a Macrocyclic Tetranuclear Complex Composed of Pt 2+ and 4,4′-Bipyridine. Chem. Lett..

[15] Orita A., Jiang L., Nakano T., Ma N., Otera J. (2002). Solventless reaction dramatically accelerates supramolecular self-assembly. Chem. Commun..

[16] Stang P.J., Cao D.H. (1994). Transition Metal Based Cationic Molecular Boxes. Self-Assembly of Macrocyclic Platinum(II) and Palladium(II) Tetranuclear Complexes. J. Am. Chem. Soc..

[17] Garci A., Castor K.J., Fakhoury J., Do J.L., Di Trani J., Chidchob P., Stein R.S., Mittermaier A.K., Friščić T., Sleiman H. (2017). Efficient and Rapid Mechanochemical Assembly of Platinum(II) Squares for Guanine Quadruplex Targeting. J. Am. Chem. Soc..

[18] Ferrer M., Pedrosa A., Rodríguez L., Rossell O., Vilaseca M. (2010). New insights into the factors that govern the square/triangle equilibria of Pd(II) and Pt(II) supramolecules. Unexpected participation of a mononuclear species in the equilibrium. Inorg. Chem..

[19] Ferrer M., Gutiérrez A., Mounir M., Rossell O., Ruiz E., Rang A., Engeser M. (2007). Self-assembly reactions between the cis-protected metal corners (N-N)M II (N-N = ethylenediamine, 4,4′-substituted 2,2′-bipyridine; M = Pd, Pt) and the fluorinated edge 1,4-bis(4-pyridyl) tetrafluorobenzene. Inorg. Chem..

[20] Rang A., Nieger M., Engeser M., Lützen A., Schalley C.A. (2008). Self-assembling squares with amino acid-decorated bipyridines: Heterochiral self-sorting of dynamically interconverting diastereomers. Chem. Commun..

[21] Zheng Y.R., Zhao Z., Wang M., Ghosh K., Pollock J.B., Cook T.R., Stang P.J. (2010). A facile approach toward multicomponent supramolecular structures: Selective self-assembly via charge separation. J. Am. Chem. Soc..

[22] Lusby P.J., Müller P., Pike S.J., Slawin A.M.Z. (2009). Stimuli-responsive reversible assembly of 2D and 3D metallosupramolecular architectures. J. Am. Chem. Soc..

[23] Iwamoto T., Watanabe Y., Sakamoto Y., Suzuki T., Yamago S. (2011). Selective and Random Syntheses of [n]Cycloparaphenylenes (n = 8–13) and Size Dependence of Their Electronic Properties. J. Am. Chem. Soc..

[24] Cui S., Zhuang G., Wang J., Huang Q., Wang S., Du P. (2019). Multifunctionalized octamethoxy-[8]cycloparaphenylene: Facile synthesis and analysis of novel photophysical and photoinduced electron transfer properties. Org. Chem. Front..

[25] Lucas F., Sicard L., Jeannin O., Rault-Berthelot J., Jacques E., Quinton C., Poriel C. (2019). [4]Cyclo-N-ethyl-2,7-carbazole: Synthesis, Structural, Electronic and Charge Transport Properties. Chem. A Eur. J..

[26] Sun Z., Sarkar P., Suenaga T., Sato S., Isobe H. (2015). Belt-Shaped Cyclonaphthylenes. Angew. Chem..

[27] Yamamoto T., Wakabayashi S., Osakada K. (1992). Mechanism of C C coupling reactions of aromatic halides, promoted by Ni(COD)_2_ in the presence of 2,2′-bipyridine and PPh_3_, to give biaryls. J. Organomet. Chem..

[28] Zheng X.H., Chen H.Y., Tong M.L., Ji L.N., Mao Z.W. (2012). Platinum squares with high selectivity and affinity for human telomeric G-quadruplexes. Chem. Commun..

[29] Kumazawa K., Biradha K., Kusukawa T., Okano T., Fujita M. (2003). Multicomponent assembly of a pyrazine-pillared coordination cage that selectively binds planar guests by intercalation. Angew. Chem. Int. Ed..

[30] Bruce M.I., Costuas K., Halet J.F., Hall B.C., Low P.J., Nicholson B.K., Skeltone B.W., White A.H. (2002). Preparation of buta-1,3-diynyl complexes of platinum(II) and their use in the construction of neutral molecular squares: Synthesis, structural and theoretical characterisation of cyclo-{Pt(μ-C≡CC≡C)(dppe)}4 and related chemistry. J. Chem. Soc. Dalton Trans..

[31] Janka M., Anderson G.K., Rath N.P. (2004). Synthesis of neutral molecular squares composed of bis(phosphine)platinum corner units and dialkynyl linkers. Solid-state characterization of [Pt(μ-C≡CC≡C)(dppp)] 4. Organometallics.

[32] Stang P.J., Fan J., Olenyuk B. (1997). Molecular architecture via coordination: Self-assembly of cyclic cationic porphyrin aggregates via transition-metal bisphosphane auxiliaries. Chem. Commun..

[33] Würthner F., Sautter A. (2000). Highly fluorescent and electroactive molecular squares containing perylene bisimide ligands. Chem. Commun..

[34] Stang P.J., Olenyuk B. (1996). Directed Self-Assembly of Chiral, Optically Active Macrocyclic Tetranuclear Molecular Squares. Angew. Chem. Int. Ed. Engl..

[35] Gupta G., You Y., Hadiputra R., Jung J., Kang D.K., Lee C.Y. (2019). Heterometallic bodipy-based molecular squares obtained by self-assembly: Synthesis and biological activities. ACS Omega.

[36] Yang H.-B., Das N., Huang F., Hawkridge A.M., Díaz D.D., Arif A.M., Finn M.G., Muddiman D.C., Stang P.J. (2006). Incorporation of 2,6-Di(4,4′-dipyridyl)-9-thiabicyclo[3.3.1]nonane into Discrete 2D Supramolecules via Coordination-Driven Self-Assembly. J. Org. Chem..

[37] Pollock J.B., Schneider G.L., Cook T.R., Davies A.S., Stang P.J. (2013). Tunable Visible Light Emission of Self-Assembled Rhomboidal Metallacycles. J. Am. Chem. Soc..

[38] Grishagin I.V., Pollock J.B., Kushal S., Cook T.R., Stang P.J., Olenyuk B.Z. (2014). In vivo anticancer activity of rhomboidal Pt(II) metallacycles. Proc. Natl. Acad. Sci. USA.

[39] Pollock J.B., Cook T.R., Stang P.J. (2012). Photophysical and Computational Investigations of Bis(phosphine) Organoplatinum(II) Metallacycles. J. Am. Chem. Soc..

[40] Chen J.-S., Zhao G.-J., Cook T.R., Han K.-L., Stang P.J. (2013). Photophysical Properties of Self-Assembled Multinuclear Platinum Metallacycles with Different Conformational Geometries. J. Am. Chem. Soc..

[41] Fan Y., Zhang J., Li Y., Chen Q., Ni Z., Zhou H., Yu J., Qiu H., Yin S. (2022). Amphiphilic rhomboidal metallacycles with aggregation-induced emission and aggregation-caused quenching luminogens for white-light emission and bioimaging. Mater. Chem. Front..

[42] Schmidtendorf M., Pape T., Hahn F.E. (2013). Molecular rectangles from platinum(ii) and bridging dicarbene, diisocyanide and 4,4′-bipyridine ligands. Dalton Trans..

[43] Stacey O.J., Platts J.A., Coles S.J., Horton P.N., Pope S.J.A. (2015). Phosphorescent, Cyclometalated Cinchophen-Derived Platinum Complexes: Syntheses, Structures, and Electronic Properties. Inorg. Chem..

[44] Kui S.C.F., Hung F.-F., Lai S.-L., Yuen M.-Y., Kwok C.-C., Low K.-H., Chui S.S.-Y., Che C.-M. (2012). Luminescent Organoplatinum(II) Complexes with Functionalized Cyclometalated C^N^C Ligands: Structures, Photophysical Properties, and Material Applications. Chem. Eur. J..

[45] Garcia M.H., Mendes P.J., Robalo M.P., Dias A.R., Campo J., Wenseleers W., Goovaerts E. (2007). Compromise between conjugation length and charge-transfer in nonlinear optical η5-monocyclopentadienyliron(II) complexes with substituted oligo-thiophene nitrile ligands: Synthesis, electrochemical studies and first hyperpolarizabilities. J. Organomet. Chem..

[46] Subach F.V., Verkhusha V.V. (2012). Chromophore Transformations in Red Fluorescent Proteins. Chem. Rev..

[47] Pollock J.B., Cook T.R., Schneider G.L., Lutterman D.A., Davies A.S., Stang P.J. (2013). Photophysical Properties of Endohedral Amine-Functionalized Bis(phosphine) Pt(II) Complexes as Models for Emissive Metallacycles. Inorg. Chem..

[48] Maroń A., Szlapa A., Czerwińska K., Małecki J.G., Krompiec S., Machura B. (2016). Solid-state and solution photoluminescence of platinum(ii) complexes with 4′-substituted terpyridine ligands—Structural, spectroscopic and electrochemical studies. CrystEngComm.

[49] Berezin M.Y., Achilefu S. (2010). Fluorescence Lifetime Measurements and Biological Imaging. Chem. Rev..

[50] Chen B.B., Li R.S., Liu M.L., Zhang H.Z., Huang C.Z. (2017). Self-exothermic reaction prompted synthesis of single-layered graphene quantum dots at room temperature. Chem. Commun..

[51] Gui R., Jin H., Bu X., Fu Y., Wang Z., Liu Q. (2019). Recent advances in dual-emission ratiometric fluorescence probes for chemo/biosensing and bioimaging of biomarkers. Coord. Chem. Rev..

[52] Huang X., Song J., Yung B.C., Huang X., Xiong Y., Chen X. (2018). Ratiometric optical nanoprobes enable accurate molecular detection and imaging. Chem. Soc. Rev..

[53] Farinola G.M., Ragni R. (2011). Electroluminescent materials for white organic light emitting diodes. Chem. Soc. Rev..

[54] Behera S.K., Park S.Y., Gierschner J. (2021). Dual Emission: Classes, Mechanisms, and Conditions. Angew. Chem. Int. Ed..

[55] Li J., Wang J., Li H., Song N., Wang D., Tang B.Z. (2020). Supramolecular materials based on AIE luminogens (AIEgens): Construction and applications. Chem. Soc. Rev..

[56] Guan J., Shen C., Peng J., Zheng J. (2021). What Leads to Aggregation-Induced Emission?. J. Phys. Chem. Lett..

[57] Zhang K., Liu J., Zhang Y., Fan J., Wang C.-K., Lin L. (2019). Theoretical Study of the Mechanism of Aggregation-Caused Quenching in Near-Infrared Thermally Activated Delayed Fluorescence Molecules: Hydrogen-Bond Effect. J. Phys. Chem. C.

[58] (2016). APEX3; SAINT, SHELXT.

[59] Sheldrick G.M. (1997). SADABS 1996.

[60] Sheldrick G.M. (2015). Crystal structure refinement with SHELXL. Acta Crystallogr. Sect. C Struct. Chem..

[61] Hübschle C.B., Sheldrick G.M., Dittrich B. (2011). ShelXle: A Qt graphical user interface for SHELXL. J. Appl. Crystallogr..

[62] Kratzert D., Krossing I. (2018). Recent improvements in DSR. J. Appl. Cryst..

[63] Spek A.L. (2015). PLATON SQUEEZE: A tool for the calculation of the disordered solvent contribution to the calculated structure factors. Acta Cryst..

