# Supplementary material for: Dual-Emissive Rectangular Supramolecular Pt(II)-p-Biphenyl with 4,4′-Bipyridine Derivative Metallacycles: Stepwise Synthesis and Photophysical Properties
Source: Molecules. 2023 Oct 25;28(21):7261. doi: 10.3390/molecules28217261 (PMC10649779; doi:10.3390/molecules28217261)
Supplement: Supplementary file 1 [file molecules-28-07261-s001.zip › molecules-2649319-supplementary.pdf]

SUPPLEMENTARY MATERIALS

Dual-Emissive Rectangular Supramolecular Pt(II)-*p*-Biphenyl with 4,4′-Bipyridine Derivative Metallacycles: Stepwise Synthesis and Photophysical Properties

Antonia Garypidou [1], Konstantinos Ypsilantis [1], John C. Plakatouras [1,2] and Achilleas Garoufis [1,2,*]

[1] Department of Chemistry, University of Ioannina, GR-45110 Ioannina, Greece; a.garypidou@uoi.gr (A.G.); k_ipsilantis@yahoo.gr (K.Y.); iplakatu@uoi.gr (J.C.P.)
[2] Institute of Materials Science and Computing, University Research Centre of Ioannina (URCI), GR-45110 Ioannina, Greece
* Correspondence: agaroufi@uoi.gr

TABLE OF CONTENTS

Figure S1: Aromatic region of 1H-1H ROESY spectrum of complex (**4**) in dmso-d_6 at 298 K in 500 MHz with assignment at interligand crosspeaks.
Table S1: Crystal data and structure refinement for $\{[Pt(2,2'\text{-bpy})]_4(\mu\text{-bph})_2(\mu\text{-}(4,4'\text{-bpy})_2\}\{PF_6\}_4$ (**8**).
Details on the refinement procedure

Figure S2: A packing diagram of compound (**8**) down to a axis of the unit cell. The void space is located within the parallelogram cations.
Figure S3: Fluorescence lifetimes for (a) (**8**), (b) (**9**) and (c) (**10**).

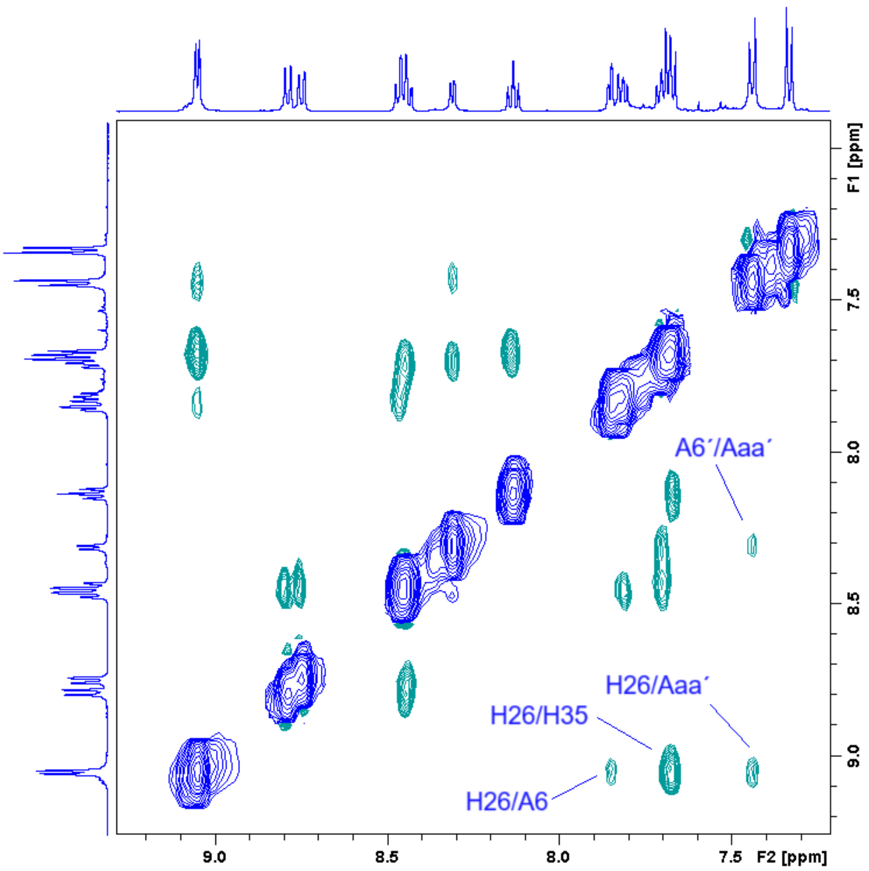

Figure S1: Aromatic region of 1H-1H ROESY spectrum of complex (**4**) in dmso-d_6 at 298 K in 500 MHz with assignment at interligand crosspeaks.

Table S1. Crystal data and structure refinement for {[Pt(2,2′-bpy)]$_4$(μ-bph)$_2$(μ-(4,4′-bpy)$_2$}{PF_6}$_4$ (**8**).

Empirical formula	$C_{84}H_{64}F_{24}N_{12}P_4Pt_4$
Formula weight	2601.71
Temperature	296(2) K
Wavelength	0.71073 Å
Crystal system	Triclinic
Space group	P-1
Unit cell dimensions	a = 11.8384(15) Å, α = 73.148(4)°<br>b = 13.3647(18) Å, β = 73.456(4)°<br>c = 17.214(3) Å, γ = 70.933(4)°
Volume	2408.6(6) Å^3
Z	1
Density (calculated)	1.794 g/cm^3
Absorption coefficient	5.950 mm^{-1}
F(000)	1240
Crystal size	0.120 x 0.060 x 0.020 mm^3

θ range for data collection	2.461 to 25.027°
Index ranges	-14<=h<=14, -15<=k<=15, -20<=l<=20
Reflections collected	37619
Independent reflections	8491 [R_{int} = 0.2353]
Completeness to θ = 25.027°	99.9 %
Refinement method	Full-matrix least-squares on F^2
Data / restraints / parameters	8491 / 560 / 617
Goodness-of-fit	0.977
Final R indices [I > 2σ(I)]	R_{obs} = 0.0750, wR_{obs} = 0.1586
R indices [all data]	R_{all} = 0.2059, wR_{all} = 0.2142
Largest diff. peak and hole	1.465 and -0.738 e·Å^{-3}

$R = \Sigma||F_o|-|F_c|| / \Sigma|F_o|$, $wR = \{\Sigma[w(|F_o|^2 - |F_c|^2)^2] / \Sigma[w(|F_o|^4)]\}^{1/2}$ and $w=1/[\sigma^2(Fo^2)+(0.0827P)^2]$ where $P=(Fo^2+2Fc^2)/3$

Details on the refinement procedure

Despite our systematic and copious attempts to grow better quality crystals, and several data set collections, the crystals obtained diffracted weakly especially at higher angles.

Our first attempts on refinement lead to several level A Platon alerts for Large Hirshfeld Differences between atoms belonging on the ligands. Our initial tries to sort out this problem involved changing the donor atoms of the bph and 4,4′-bpy ligands and eventually treating all ligands as disordered. This lead systematically to several atoms with non-positive-definite. Those alerts were removed utilizing SIMU restraints.

The rest of the B alerts (i.e. The value of Rint is greater than 0.18, Ratio Observed / Unique Reflections (too) Low .. 37% and Low Bond Precision on C-C Bonds) kept insisting because of the disordering present in the structure, which can make the diffracted intensity fall very sharply with diffracting angle.

One of the PF_6^- anions is severely disordered. It was modeled with the assistance of DSR [1] in two positions with occupancies approximately 0.47 and 0.53.

There is also some residual electron density in the crystal structures which could not be modeled. It assigned to solvated molecules and it was treated with Platon's subroutine SQUEEZE [2]. Eventually that lead to 329 Å^3 per unit cell void space which contained 50 electrons. The number of electrons corresponds very well to approximately 2,5 molecules of MeCN per unit cell.

Despite the crystallographic problems, the molecular structure model is chemically sound, with geometrical characteristics similar to other published compounds (see references in the article) and is in agreement with other spectroscopic and physicochemical results.

References

[1] D. Kratzert, I. Krossing, J. Appl. Cryst.(2018).51, 928–934, doi: 10.1107/S1600576718004508

[2] A. L. Spek, Acta Cryst. (2015). C71, 9–18, doi:10.1107/S2053229614024929

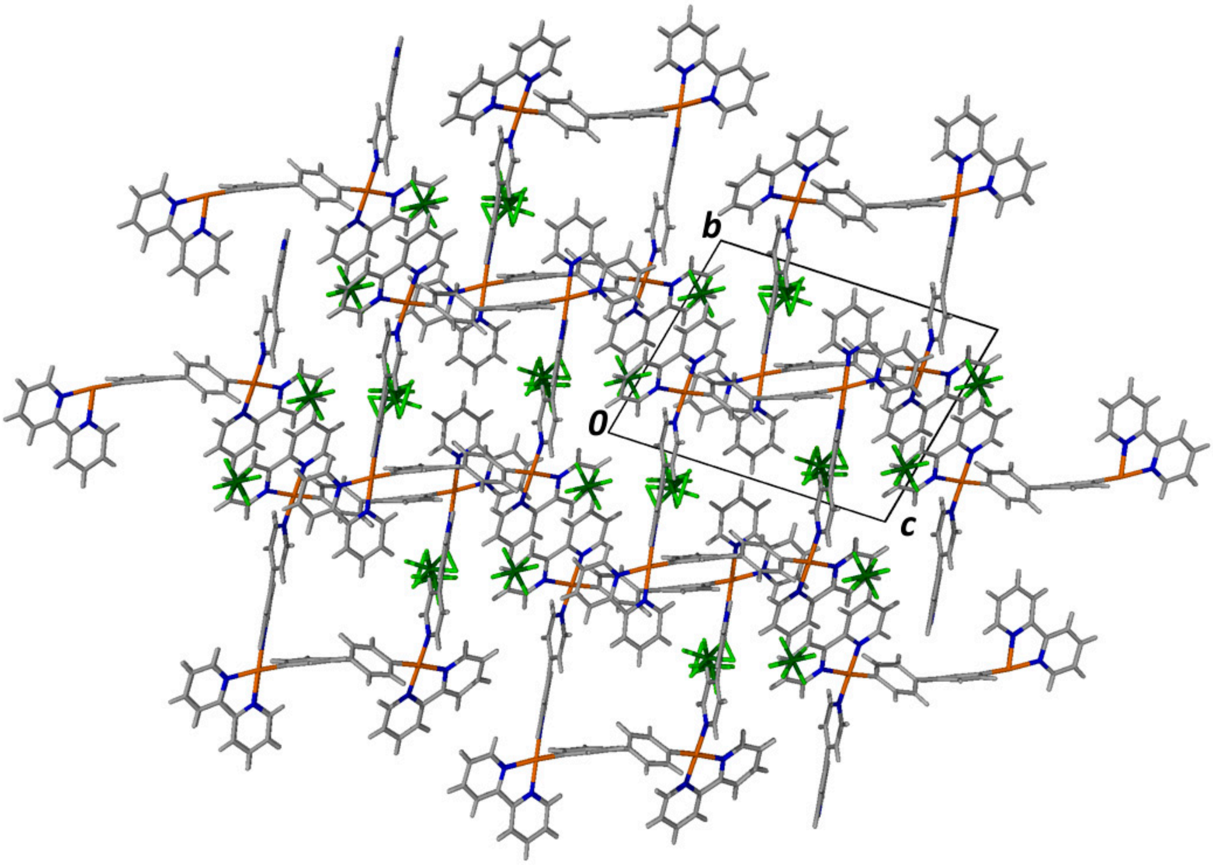

Figure S2: A packing diagram of compound (**8**) down to a axis of the unit cell. The void space is located within the parallelogram cations.

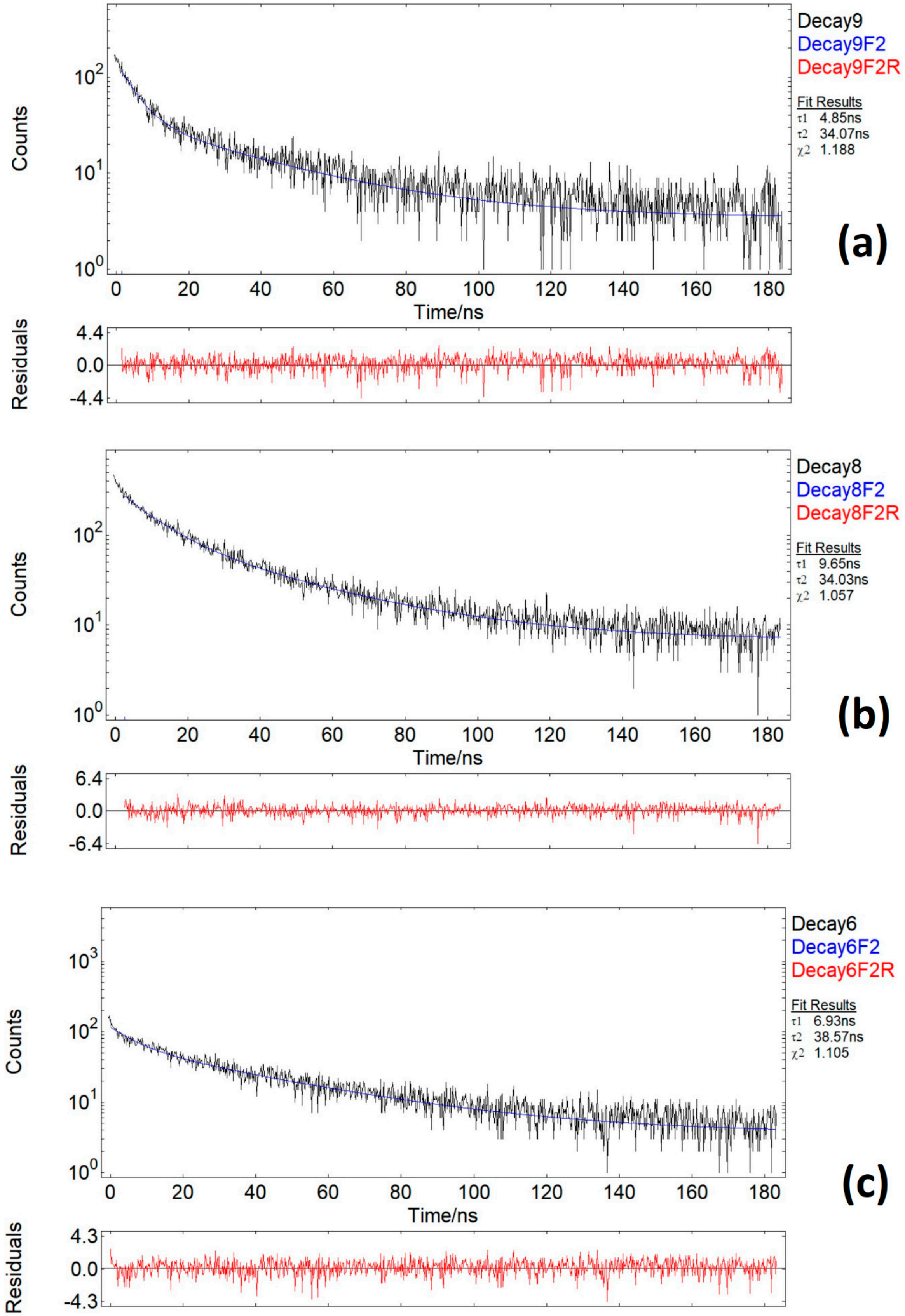

Figure S3: Fluorescence lifetimes for (a) (**8**), (b) (**9**) and (c) (**10**).
